# Erythrocyte Membrane Fluidity and Omega-3 Fatty Acid Intake: Current Outlook and Perspectives for a Novel, Nutritionally Modifiable Cardiovascular Risk Factor

**DOI:** 10.3390/nu16244318

**Published:** 2024-12-14

**Authors:** Umberto Capece, Shawn Gugliandolo, Cassandra Morciano, Adriana Avolio, Amelia Splendore, Gianfranco Di Giuseppe, Gea Ciccarelli, Laura Soldovieri, Michela Brunetti, Teresa Mezza, Alfredo Pontecorvi, Andrea Giaccari, Francesca Cinti

**Affiliations:** 1 Endocrinologia e Diabetologia, Fondazione Policlinico Universitario Agostino Gemelli IRCCS, 00136 Rome, Italy; 2 Dipartimento di Medicina e Chirurgia Traslazionale, Università Cattolica del Sacro Cuore, 00168 Rome, Italy

**Keywords:** residual cardiovascular risk, omega-3, omega-6, EPA, DHA, erythrocyte membrane fluidity, RBC

## Abstract

Omega-3 fatty acids reduce triglycerides and have several positive effects on different organs and systems. They are also found in the plasma membrane in variable amounts in relation to genetics and diet. However, it is still unclear whether omega-3 supplementation can reduce the occurrence of major cardiovascular events (MACEs). Two trials, REDUCE-IT (Reduction of Cardiovascular Events with Icosapent Ethyl-Intervention Trial), with highly purified EPA, and STRENGTH (Effect of High-Dose Omega-3 Fatty Acids vs. Corn Oil on Major Adverse Cardiovascular Events in Patients at High Cardiovascular Risk), with a combination of EPA and DHA, have produced different outcomes, triggering a scientific debate on possible explanations for the discrepancies. Furthermore, doubts have arisen as to the anti-inflammatory and anti-aggregating activity of these compounds. Recent studies have, however, highlighted interesting effects of EPA and DHA on erythrocyte membrane fluidity (EMF). EMF is governed by a complex and dynamic biochemical framework, with fatty acids playing a central role. Furthermore, it can be easily measured in erythrocytes from a blood sample using fluorescent probes. Recent research has also shown that EMF could act as a possible cardiovascular risk factor biomarker. This review aims to synthetize the latest evidence on erythrocyte membrane fluidity, exploring its potential role as a biomarker of residual cardiovascular risk and discussing its clinical relevance. Further, we aim to dissect the possible biological mechanisms that link omega-3 modifiable membrane fluidity to cardiovascular health.

## 1. Introduction

Dietary intake and supplementation with omega-6 and omega-3 polyunsaturated fatty acids (PUFAs) is the subject of intense and ongoing scientific debate. Several studies have demonstrated that these molecules have a broad spectrum of positive effects [1,2,3], but controversies have emerged as to their real necessity and functions [4]. Overall, evidence for the beneficial effects of omega-3 appears more robust than that for omega-6 [5,6], but recently, the continuous intake of linoleic acid (LA), an omega-6 PUFA, has been associated with reduced all-cause mortality [7]. Further, omega-3 supplementation has been established as a valid option for the treatment of hypertriglyceridemia [8], but clinical evidence in favor of increased intake for the treatment of cardiovascular diseases and the prevention of CV events is still weak [9].

Recently, the “Reduction of Cardiovascular Events with Icosapent Ethyl-Intervention Trial” (REDUCE-IT) demonstrated, for the first time, that omega-3 treatment with highly purified eicosapentaenoic acid (EPA) reduced cardiovascular events in patients with established cardiovascular disease or diabetes and other risk factors. The enrolled patients had mild hypertriglyceridemia (≥150 mg/dL and <500 mg/dL) and were on stable statin ± ezetimibe treatment [10]. Interestingly, since benefits were seen regardless of baseline and post-treatment triglyceride levels, the effects of EPA on cardiovascular risk could be due not only to triglyceride reduction, but perhaps also to an anti-inflammatory effect.

However, despite a nonspecific reduction in C-reactive protein and oxidized low-density lipoprotein (Ox-LDL), there are still uncertainties as to a possible effect on adhesion molecules and IL-6 levels [11]. EPA also competes with arachidonic acid for the binding of cyclooxygenase (COX), leading to the formation of mediators with lower platelet-aggregating capacity [12]. However, the antiaggregant effect of EPA also appears to be controversial since, in a randomized-controlled trial in healthy young males, no effect on platelet–monocyte aggregation was documented [13]. Finally, Sherratt et al. [14,15] found that EPA and docosahexaenoic acid (DHA) had contrasting effects on plasma membrane fluidity, with EPA reducing fluidity and DHA increasing fluidity. This provides a possible explanation for the contrasting results obtained by the REDUCE-IT trial compared to the STRENGTH Randomized Clinical Trial (Effect of High-Dose Omega-3 Fatty Acids vs. Corn Oil on Major Adverse Cardiovascular Events in Patients at High Cardiovascular Risk) [16]. In the latter, combined therapy with EPA and DHA failed to reduce the primary composite cardiovascular endpoint. Thus, beyond the triglyceride-lowering effect, increased membrane stability and, consequently, reduced fluidity may be the most plausible explanation for the benefits yielded by EPA compared to DHA and for the different results obtained by the REDUCE-IT and the STRENGTH trials.

The red blood cell (RBC) membrane is the easiest cell membrane to evaluate, as it can be assessed from a blood sample. Furthermore, since the RBC lacks a nucleus and cannot synthesize proteins, the features of the RBC membrane largely reflect the state of systemic metabolism [17]_._ Recently, our group demonstrated that increased erythrocyte membrane fluidity (EMF) is associated with a higher CV risk in subjects with type 2 diabetes (T2D) [18]. Thus, the biophysical properties of the membrane could be linked to the occurrence of cardiovascular diseases, making EMF a possible cardiovascular risk factor.

The concept of “risk factor” in multifactorial diseases is as paramount as causative agents are for infectious diseases [19].

Risk factor management is a crucial aspect in reducing the likelihood of a cardiovascular event [20]. However, even an optimal control of known risk factors leaves what is generally defined as “residual cardiovascular risk” [21]. Thus, over time, novel lipid biomarkers have been identified to detect further risk [22]. In this context, triglycerides have generated significant interest. The association between elevated triglyceride levels and CV event risk [23,24,25] is supported by observational studies and clinical trials; however, except for EPA in REDUCE-IT [10], studies investigating triglyceride-lowering interventions have shown disappointing results on CV outcomes [16,26].

The aim of this review is to report the latest evidence on EMF, discuss its potential role as a novel CV risk factor and its clinical relevance, and describe the possible biological mechanisms that link erythrocyte membrane fluidity to cardiovascular health.

## 2. From the Fluid Mosaic Model to Erythrocyte Membrane Fluidity

In 1972, Singer and Nicholson were the first to describe the fluid mosaic model of the structure of the cell membrane: a dynamic structure made up of phospholipids organized as a discontinuous fluid bilayer in which amphipathic integral proteins are disposed with the polar groups protruding from the membrane into the aqueous phase and the nonpolar groups in the hydrophobic interior of the membrane [27]. When the temperature increases, the membrane moves from the gel phase to the fluid phase. The transition temperature between the two phases depends on the type of phospholipid within the membrane. By increasing the degree of desaturation of membrane fatty acids, the transition temperature between the gel-like state and the fluid phase decreases, guaranteeing the fluid phase even in lower temperatures [28]. Cold-water marine animals have higher levels of unsaturated and omega-3 fatty acids compared to warm fresh-water species [29]. In fact, cells respond to low temperatures by changing the composition of their membrane phospholipids through the activity of enzymes such as “desaturases” and “phospholipases”, which can increase the amount of unsaturated fatty acids [30]. Conversely, the presence of saturated fatty acids allows the phospholipids to thicken in a more compact manner. Chain length also affects fluidity: phospholipids containing long-chain fatty acids (i.e., with 13–21 carbon atoms) tend to pack together, giving the membrane a more solid, gel-like structure [31].

Red blood cells (RBCs) are the most prevalent cells in the body (about 5 × 10^6^ cells/mm^3^ of blood with an average life span of 120 days) [32]. They have a skeleton which determines the peculiar shape of the cell and allows for its high deformability [33]. This flexible membrane is associated with other unique structural features such as the lack of a nucleus, mitochondria, and ribosomes. For these reasons, RBCs cannot synthesize amino acids and fatty acids and have a limited metabolism capacity, which is barely enough to cover their life span [34]. In brief, pluripotent hematopoietic stem cells generate erythroid precursors (erythroblasts), which give birth to reticulocytes after ejecting their nuclei [35,36]. Reticulocytes lose their organelles and generate erythrocytes. Protein band 3 and glycophorin A are the most expressed integral membrane proteins [37], while hemoglobin represents more than 95% of cytoplasmic proteins [38], and, beyond its role in oxygen transport, this protein could be involved in ageing, erythrocyte metabolism, and many other functions [39]. Despite its simple composition, increasing evidence links erythrocyte metabolism with the pathogenesis of atherosclerosis [40,41]. The mechanisms responsible for this close connection are several: first, during hemolysis RBC release free hemoglobin to the plasma that acts as a potent nitric oxide scavenger [42]. Secondly, intraplaque hemorrhage is common in advanced atherosclerotic lesions and leads to the accumulation of free cholesterol from erythrocyte membranes [43]. Third, microvesicles derived from erythrocytes have been proven to promote atherosclerosis by inducing hypercoagulation and cell adhesion and mediating inflammation [44].

This close connection between erythrocyte metabolism and atherosclerosis becomes even more pronounced in diabetes. Chronic hyperglycemia leads to the glycation of many proteins, including hemoglobin glycation (HbA1c), which has been extensively studied [45]. HbA1c represents the main glucose control indicator in clinical practice [46], but structural proteins and enzymes are also glycated in diabetes, leading to the production of advanced glycation end products (AGE), which are involved in many diabetic complications [40,47]. In addition, erythrocyte deformability is compromised due to Protein Band 3 oxidative damage [48] with subsequent increased membrane aggregation [49]. Thus, in diabetes, the erythrocyte membrane is damaged due to the overlap of various pathophysiological processes.

Cholesterol is another primary component of the membrane: it has a “stabilizing” role in relation to the membrane’s phase. In membranes in the fluid phase, it tends to compact unsaturated fatty acid-rich phospholipids, while in the gel phase, it makes the double layer more fluid, reducing the interaction between long-chain fatty acids and saturated fatty acids [50]. RBCs do not express the LDL receptor; therefore, membrane cholesterol content largely depends on systemic lipoprotein metabolism [51]. The sphingomyelin content and lecithin cholesterol acyltransferase activity of the erythrocyte membrane regulate the transfer rate of free cholesterol between the cell membrane and plasma lipoproteins [52]. However, the relationship between erythrocyte membrane cholesterol (EMC) and lipoproteins remains unclear, as only a slight and non-significant increase in EMC level was found in dyslipidemic subjects compared to normolipidemic subjects, with a significant negative correlation with small-dense LDL [53]. Treatment with statins has shown controversial results regarding this parameter [54,55]. Conversely, an RCT showed that erythrocyte fatty acid content can be easily modified by omega-3 supplementation in a dose-dependent manner [56].

Since fatty acid content determines the arrangement of phospholipids in the fluid mosaic model of RBC, increasing it could be a promising and easily achievable method to resolve membrane impairments.

Translating the biological complexity of the erythrocyte membrane into a physical parameter appears to be rather complex. Several methods that measure EMF from different perspectives can be found in the literature, and these different methods could also explain the discrepancies in the observed results pertaining to EMF in specific populations. The lipophilic index (LI) mainly reflects fatty acid fluidity and is obtained by summing the products of fatty acid levels and the melting point of each [57]. EMF measurement techniques consist of the dilution of the blood with a specific fluorescent probe and the subsequent evaluation of the fluorescence obtained following erythrocyte excitation. 1,6-diphenyl-1,3,5-hexatriene (DPH) is a hydrophobic probe that measures viscosity at the level of the fatty acid chains of phospholipids [58]; on the other hand, 2-dimethylamino-6-lauroylnaphthalene (Laurdan) is disposed at the hydrophilic–hydrophobic membrane interface [59].

In conclusion, maintaining the physiological integrity of the erythrocyte fluid mosaic model and metabolism could be an intriguing objective for the prevention and management of cardiovascular complications.

## 3. Erythrocyte Membrane Fluidity in Human Physiology

Since EMF is the outcome of a highly dynamic process involving different components of the membrane, numerous factors can influence this parameter. In this paragraph, we will describe how modifications of membrane components such as cholesterol and phospholipids can influence the biochemical properties of the membrane in the absence of disease. Tziakas et al. proposed the hypothesis that erythrocyte membrane cholesterol (EMC) could provide insights into the long-term trends of the lipid profile [52], as it appears unaffected by acute fluctuations in cholesterol levels and low-lipid diets; however, no significant differences have been found between normocholesterolemic and hypercholesterolemic subjects [53]. On the other hand, phospholipids have a central role in the membranes of all living species, and recently, much attention has been devoted to their antioxidant and pro-oxidant mechanisms [60]. The term “phospholipid” generically refers to a molecule which comprises a phosphatidic group, glycerol, and fatty acids. A more appropriate chemical classification distinguishes glycerophospholipids (which are more represented at the membrane level) from sphingolipids, both characterized by the presence of fatty acids [61]. These molecules can change their composition over time in response to different diets, as assessed in the EPIC study [62]. Thus, phospholipids, rather than cholesterol, could be the main protagonists of changes in EMF.

Glycerophospholipids can exhibit different fatty acids, such as MUFA, omega-6 PUFA, and omega-3 PUFA, that reflect dietary habits: for example, the MUFA oleic acid increases with olive oil consumption, while long-chain omega-3 fatty acids in plasma phospholipids are associated with fish consumption [62]. Trans MUFAs originate as a result of partial hydrogenation (reduction in some unsaturation with conversion of cis- to trans-unsaturation) and have consequences on CV risk as they increase LDL-C [63].

Erythrocyte fatty acid content levels has been shown to correlate with dietary intake more than serum fatty acids; in particular, this superiority appears more robust for erythrocyte omega-3 fatty acids of marine origin and trans fatty acids [64]. Conversely, an omega-6 rich diet induces similar alterations in plasma and erythrocyte fatty acid composition over a comparable time frame [65]. The relationship between omega-3 intake and erythrocyte membrane fatty acids (EMFA) has been extensively explored, particularly in terms of EPA + DHA supplementation, in which a dose-dependent increase in these fatty acids in RBC was observed: EPA comprised up to 2.46 % of erythrocyte weight in the group treated with EPA + DHA at the maximum dose (1800 mg/day), 0.96% in the group treated with the lowest dose, and 0.47% in the placebo group, while DHA reached 7.03%, 5.3%, and 3.87%, respectively [56]. Elderly and female subjects exhibited larger variations in EMFA content. Moreover, the administration of intravenous omega-3 PUFAs has been shown to lead to rapid modifications in EMFA levels [66]. Omega-3 FAs can be found in various sources, such as fish, nuts, and seeds [67].

Numerous dietary interventions have validated the capacity of diets to influence EMFA content [68,69,70]. However, beyond diet and supplementation, genetic factors could be involved as well. Two genes have attracted great interest: fatty acid desaturase 1 (FADS1), which encodes for Δ-5 desaturase, and fatty acid desaturase 2 (FADS2), which encodes for Δ-6 desaturase, both localized on chromosome 11 [71,72]. These enzymes are rate-limiting in several biochemical reactions, such as the desaturation of linoleic acid (LA) to arachidonic acid (AA) and that of alpha-linolenic acid (ALA) to EPA and DHA. Polymorphisms in these genes have been described, associated with lower levels of AA (the main precursor of eicosanoids) and EPA [72]. AA levels exhibit higher genetic variation than EPA. In the Verona Heart Project, which included 658 adults, 13 SNPs were discovered, characterized by higher levels of LA and ALA and lower levels of AA, without changes in EPA or DHA in phospholipids and the erythrocyte membrane [73]. It is likely that the EPA and DHA content in RBCs is mainly determined by diet, while the genetic predisposition is significant for AA content. Further, EPA and DHA supplementation increase Δ-5 desaturase activity and decrease Δ-6 desaturase activity [74]. Based on these data, EMF appears to depend on both diet and genetic factors, and omega-3 fatty acids seem to play a central role.

## 4. Possible Biochemical Mechanisms Leading to Erythrocyte Membrane Changes

Certain dietary factors can negatively impact membrane fluidity, in particular, trans-MUFAs, as they not only raise LDL-C cholesterol levels but also tend to pack together more easily than cis-MUFAs. The latter are prevented from close packing by the presence of a “kink” in the fatty acid chain [75].

Furthermore, EPA and DHA have aroused great interest in the scientific community due to their distinct effect on the chemical and physical properties of the membrane, despite their similar chemical structures and metabolism. These compounds both belong to the omega-3 group, but DHA has six double bonds, whereas EPA has five [76]. EPA can be found in algae such as *Undaria Pinnafitida* and in the muscle tissues of several fish, fish roe, and sardines, while the main sources of DHA are flying fish, herring, pollock, and salmon roe [67]. It has been demonstrated that EPA enhances membrane electron density within a region extending 10 A° from the center, while DHA increases the electron density at the level of the hydrophilic heads [14]. Moreover, their interaction with membrane cholesterol presents disparities in an experimental model: under low-cholesterol conditions, both EPA and DHA reduce the energy associated with membrane stretching; when cholesterol increases, EPA increases this parameter, while DHA does not [15]. This different behavior could reflect a distinct interaction with cholesterol. EPA interacts with the latter by forming ordered raft-like phases, whereas DHA’s conformation prompts the formation of disordered crystalline domains [15,77]. It is likely that this different arrangement affects the transition to GEL phase and, consequentially, fluidity. The interaction between phospholipids and cholesterol emerges as a pivotal factor, particularly in conditions favoring hyperglycemia, where the formation of these domains is exacerbated. Notably, EPA also inhibits glucose-induced cholesterol domain formation [78].

Thus, the possibility of modulating EPA levels through diet or supplementation represents an intriguing method to improve erythrocyte membrane arrangement, and these notions could explain the different results between REDUCE-IT [10] and STRENGTH [16].

Finally, a recent study showed that fish consumption leads to a reduction in the lipophilic index in the erythrocyte membrane that is inversely correlated with HDL diameter [79]. Thus, HDL particles could indirectly affect EMF by reducing oxidative stress after an omega-3 trigger [80]. Other unknown biochemical mechanisms could be involved in the pathophysiology of EMF, which is a new research area. While we have gained substantial insights into the lipid composition of the erythrocyte membrane, understanding how interactions among these substances affect the membrane’s physical properties, including fluidity, requires further analysis. Impairments in EMF have been documented in a limited number of studies involving patients with clinically manifest diseases, such as coronary artery disease (CAD) and diabetes, which can affect membrane composition and function, as we will discuss in the next paragraph. Currently, it remains unclear whether the described EMF alterations are a cause or a consequence of these pathologies. In fact, several mechanisms link rheological impairments with ischemia onset [81], whether in the presence [82] or absence [83] of diabetes. Thus, dysfunctional EMF could alter the rheological characteristics of RBC, potentially reducing oxygen supply to peripheral tissues [84]. This suggests that EMF impairments could be the primum movens predisposing individuals to cardiovascular diseases, with the latter being only a consequence. However, given the current evidence, this remains an open question. In the next paragraph, we will discuss conditions associated with an already manifested alteration of membrane fluidity and its relationship with cardiovascular risk.

## 5. Erythrocyte Membrane Fluidity and Cardiovascular Risk

**Figure 1:**
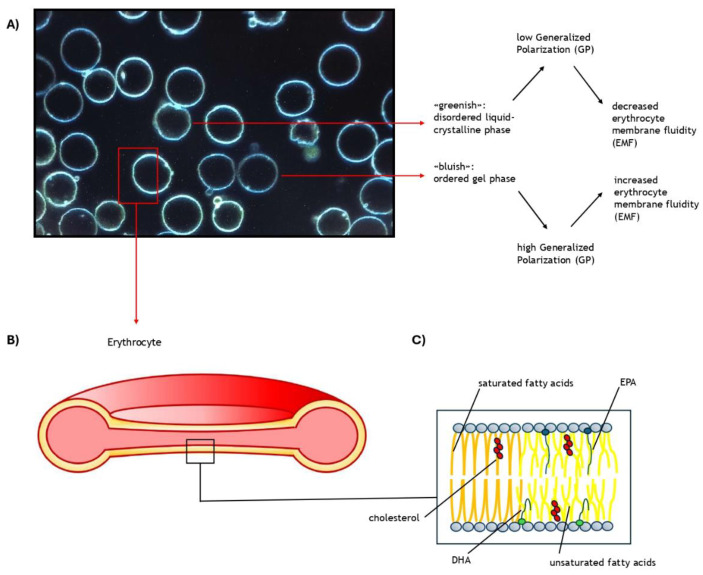
This figure is a comprehensive illustration of the latest evidence on erythrocyte membrane fluidity: (**A**) This image was obtained through confocal microscopy after staining a blood sample with a specific probe (Laurdan). This probe is characterized by a shift in the emission spectrum reflecting the lipid phase state of the environment (bluish in ordered gel phases and greenish in disordered liquid–crystalline phases). After staining, five images of Laurdan emission intensity were acquired concurrently in two distinct channels (emission filter: 450/50 nm for the blue channel, 525/50 nm for the green channel) using a 60× immersion-oil objective. Generalized polarization (GP) was calculated as follows: GP = Iblue − G Igreen/Iblue + G Igreen, where Iblue and Igreen are the intensities for the blue and green channels, respectively, and G is a calibration factor that depends on the experimental setup. GP is a measure of membrane fluidity. (**B**) Longitudinal section of an erythrocyte. (**C**) Description of the main components of the erythrocyte membrane.

Given the relationship between RBC and atherosclerosis and the pivotal role of the RBC membrane in the chemical and physical properties of blood rheology, EMF should be considered as a CV risk factor. The first human studies on the topic demonstrated increased membrane fluidity in patients affected by ischemic stroke and hypertension [85,86]. Conversely, a prospective study highlighted a more rigid hydrophobic region in survivors of acute myocardial infarction compared to controls [87]. A further rigidification of the membrane was documented during the study and after 60 months in some patients who had another cardiovascular event [88]. The authors found that baseline EMF was an independent predictor of events (*p* < 0.05). In line with these findings, a cross-sectional study has shown that patients with coronary artery disease (CAD) display an increased EMC and decreased EMF, as assessed by DPH [89]. According to the authors, increased EMC, together with increased lipid peroxidation, assessed by reduced activity of superoxide dismutase (SOD) and catalase, could cause rigidification of the hydrophobic portion of the membrane. In all these studies, membrane lipid fluidity was determined by 1,6-diphenyl-1,3,5-hexatriene (DPH). Bordoni et al., in a large cohort of CAD patients, demonstrated that EMF was decreased only in males when assessed using DPH, while no differences were observed with Laurdan [90]. This suggests differing behavior between the two probes in this clinical context. Recently, our group demonstrated that T2D patients with more fluid membranes, as evaluated with Laurdan (low general polarization), had increased odds of a macrovascular complications of diabetes (*p*  = 0.045) compared to T2D subjects with less fluid membranes (high general polarization) [18] (Figure 1). Moreover, in a logistic regression analysis, high membrane fluidity was an independent predictor of macrovascular complications, and general polarization improved the performance of the UKPDS score in classifying patients according to the presence/absence of cardiovascular diseases.

Several factors should be considered when addressing the discrepancies in the comparison of these studies (study design and features are listed in Table 1). First, our cohort evaluated T2D patients, and other macrovascular complications besides CAD were included (i.e., stroke and peripheral artery disease). Then, DPH and Laurdan provided different information about membranes in CAD as well as in the context of diabetes. In fact, previous studies have shown higher erythrocyte microviscosity (the opposite of fluidity) when assessed with DPH [91], and a higher EMF with Laurdan [92].

Diabetes entails the glycation of integral proteins of the erythrocyte membrane and increases the content of saturated fatty acids [93]. Further, high glucose levels are associated with the production of cholesterol crystalline domains in an experimental model of the cell membrane [78]. Thus, diabetes affects both membrane structure and composition. In this context, Laurdan may provide information about the overall order and fatty acid composition of the membrane. In fact, the low-general-polarization patients in our study also had increased omega-6/omega-3 ratios and decreased omega-3/PUFA indexes compared to the high-GP and CTRL groups. This confirms the presence of an association between EMF and EMFA content. However, since the study is cross-sectional, it is impossible to determine the causes of different omega-3 contents of the membrane in this cohort of patients. As we discussed earlier, the latter depends mainly on food intake and partly on genetic factors. If we presume that EMFA depends on the patients’ diet, it could be hypothesized that omega-3 decreases cardiovascular risk by improving EMF rather than by reducing triglyceridemia. Although this concept is only hypothetical, as there is insufficient data to support it, if this were the case, EMF would become a biomarker of residual cardiovascular risk which could be effectively treated with omega-3 supplementation, a simple and safe therapy.

This feature is unique in the field of lipidology. Other emerging lipid biomarkers associated with residual cardiovascular risk, such as lipoprotein (a), currently lack accessible, widespread, and effective treatments. Although several lipoprotein (a)-targeted therapies are under clinical development, they are not yet available for widespread use [94].

## 6. Erythrocyte Membrane Fluidity as a CV Risk Factor in Clinical Practice: Pros and Cons, Unmet Needs

From a lipid-centered perspective, “residual cardiovascular risk” is defined as a MACE that occurs despite statin therapy [95]. Beyond LDL-C (the main target of statins), this concept also refers to the management of the other conventional CV risk factors.

Despite significant progress in understanding cardiovascular diseases since the early days of the Framingham study, there is still a considerable knowledge gap, and novel factors could account for residual risk. In Type 2 diabetes, even when all traditional risk factors are treated intensively together, the rate of events is non-negligible and the unexplained risk could reach 44.1% [96]. Understanding the causes of such events is, thus, a matter of great relevance. In the era of precision medicine, there is increasing emphasis on utilizing biomarkers to better stratify individuals with the same condition to personalize diagnosis and therapy [97]. Further, biomarkers could also provide information about the remaining risk and follow-up after treatments [98]. While there have been considerable advancements in cardiovascular pharmacotherapy [99], omega-3 supplementation remains a relevant option in dyslipidemia treatment [100], with additional beneficial effects on cardiovascular health [101]. In the REDUCE-IT trial [10], highly purified EPA reduced cardiovascular events regardless of triglyceride levels. Thus, other mechanisms are being explored to explain these results. Moreover, the nutritional approach has several applications in cardiovascular medicine, and omega-3-rich diets have shown important results [102,103].

The wealth of data on the potential modification of erythrocyte membrane fatty acids (EMFA) through diet (including the interaction with EMC) demonstrates the effectiveness of a simple intervention in achieving significant outcomes. To date, however, no study has demonstrated the effectiveness of a dietary treatment in modulating membrane fluidity. The latter, rather than the omega-3 content, seems to be an ideal parameter to represent the complexity of the membrane and its biophysical characteristics. In modern medicine, the term risk factor includes both causal and predictive factors. The predictive value of EMF has been proven in T2D by our group [18] and by Saldanha et al. in myocardial infarction survivors [87]. However, further studies are needed to establish causality. Additionally, a new risk factor should have good sensitivity and specificity and be feasible and widely applicable in clinical practice [104]. Currently, however, the evaluation of EMF is limited to research laboratories, and uncertainties persist regarding the optimal methodology for accurate measurement. Therefore, both interventional and prospective studies should be encouraged to evaluate the response of EMF to omega-3 treatment and to establish its causal relationship with cardiovascular events. Further, methodological and cost-effectiveness studies comparing the fluorescent probes are needed to establish the most accurate and feasible methodology for clinical practice.

In conclusion, EMF represents an intriguing parameter that could offer important health insights. It is moreover linked to EMFA and can thus be effectively modified by diet. Future research is needed to validate EMF as a cardiovascular risk factor and to investigate whether the action of omega-3 on residual cardiovascular risk may primarily involve EMF.

## 7. Conclusions

The red blood cell membrane performs numerous vital functions in the body, and its dysfunctions could impact rheology and have consequences in the context of atherosclerosis. Among various parameters, membrane fluidity has aroused significant interest in the scientific community, particularly after the REDUCE-IT trial’s results. In fact, the improvement in membrane fluidity appears to be a reasonable explanation for the benefits induced by Icosapent-Ethyl. EMF is linked to EMFA, which, in turn, depends on omega-3 dietary intake. According to the above-mentioned studies, among omega-3 PUFAs, EPA improves fluidity, whereas DHA does not. One possible hypothesis to explain the lack of significant outcomes in the STRENGTH trial compared to REDUCE-IT is that the former administered a combination of EPA and DHA, while the latter used only EPA.

EMF has proven to be a predictor of cardiovascular events, a feature in common with the well-known cardiovascular risk factors of the Framingham study. However, much work is still required to determine the optimal methodology for its assessment, to promote its application, and to establish a causal relationship with the onset of atherosclerosis in order to validate EMF as a novel cardiovascular risk factor.

## Figures and Tables

**Table 1:** EMF and Cardiovascular Diseases.

Authors/Years	Country/Sample Number	Clinical Context	Study Design	Probe	Main Results
Li et al./1989 [85]	China/21 patients	Ischemic Stroke	cross-sectional	DPH	increased EMF
Miller et al./1994 [86]	United Kingdom/81 patients	Hypertension	cross-sectional	DPH	increased EMF
Saldanha et al./1999 [87,88]	Portugal/60 patients	Survivors of an acute myocardial infarction	prospective	DPH	decreased (EMF
Pytel et al./2013 [89]	Poland/20 patients	CAD	cross-sectional	DPH	decreased EMF
Bordoni et al./2020 [90]	Poland/547 patients	CAD	cross-sectional	DPH Laurdan	decreased EMF only in men no changes
Bianchetti et al./2023 [18]	Italy/234 patients	T2D	cross-sectional	Laurdan	increased EMF in patients with macrovascular complications

Studies evaluating EMF as a possible cardiovascular risk factor in the literature.

## Abbreviations

PUFA	polyunsaturated fatty acid
CHD	coronary heart disease
CVD	cardiovascular disease
EPA	eicosapentaenoic acid
DHA	docosahexaenoic acid
COX	Cyclooxygenase
RBC	red blood cell
EMF	erythrocyte membrane fluidity
EMC	erythrocyte membrane cholesterol
EMFA	erythrocyte membrane fatty acids
T2D	type 2 diabetes
AGE	advanced glycation end products
DPH	1,6-diphenyl-1,3,5-hexatriene
SFA	saturated fatty acids
PUFA	polyunsaturated fatty acids
MUFA	monounsaturated fatty acids
FADS	fatty acid desaturase
MACE	major cardiovascular events
